# Chronic pneumonia in calves after experimental infection with *Mycoplasma bovis *strain 1067: Characterization of lung pathology, persistence of variable surface protein antigens and local immune response

**DOI:** 10.1186/1751-0147-54-9

**Published:** 2012-02-04

**Authors:** Kathrin Hermeyer, Inka Buchenau, Anne Thomasmeyer, Berit Baum, Joachim Spergser, Renate Rosengarten, Marion Hewicker-Trautwein

**Affiliations:** 1 Department of Pathology, University of Veterinary Medicine Hannover, Bünteweg 17, D-30559 Hannover, Germany; 2 Institute of Bacteriology, Mycology and Hygiene, University of Veterinary Medicine Vienna, Veterinärplatz 1, A-1210 Vienna, Austria

**Keywords:** Cattle, *Mycoplasma bovis*, pneumonia, immunoglobulins, CD4^+ ^T cells, CD8^+ ^cells, MHC class II

## Abstract

**Background:**

*Mycoplasma bovis *is associated with pneumonia in calves characterized by the development of chronic caseonecrotic lesions with the agent persisting within the lesion. The purposes of this study were to characterize the morphology of lung lesions, examine the presence of *M. bovis *variable surface protein (Vsp) antigens and study the local immune responses in calves after infection with *M. bovis *strain 1067.

**Methods:**

Lung tissue samples from eight calves euthanased three weeks after experimental infection with *M. bovis *were examined by bacteriology and pathology. Lung lesions were evaluated by immunohistochemical (IHC) staining for wide spectrum cytokeratin and for *M. bovis *Vsp antigens and pMB67 antigen. IHC identification and quantitative evaluation of CD4^+ ^and CD8^+ ^T lymphocytes and immunoglobulin (IgG1, IgG2, IgM, IgA)-containing plasma cells was performed. Additionally, expression of major histocompatibility complex class II (MHC class II) was studied by IHC.

**Results:**

Suppurative pneumonic lesions were found in all calves. In two calves with caseonecrotic pneumonia, necrotic foci were surrounded by epithelial cells resembling bronchial or bronchiolar epithelium. In all calves, *M. bovis *Vsp antigens were constantly present in the cytoplasm of macrophages and were also present extracellularly at the periphery of necrotic foci. There was a considerable increase in numbers of IgG1- and IgG2-positive plasma cells among which IgG1-containing plasma cells clearly predominated. Statistical evaluation of the numbers of CD4^+ ^and CD8^+ ^T cells, however, did not reveal statistically significant differences between inoculated and control calves. In *M. bovis *infected calves, hyperplasia of bronchus-associated lymphoid tissue (BALT) was characterized by strong MHC class II expression of lymphoid cells, but only few of the macrophages demarcating the caseonecrotic foci were positive for MHC class II.

**Conclusions:**

The results from this study show that infection of calves with *M. bovis *results in various lung lesions including caseonecrotic pneumonia originating from bronchioli and bronchi. There is long-term persistence of *M. bovis *as demonstrated by bacteriology and immunohistochemistry for *M. bovis *antigens, i.e. Vsp antigens and pMB67. The persistence of the pathogen and its ability to evade the specific immune response may in part result from local downregulation of antigen presenting mechanisms and an ineffective humoral immune response with prevalence of IgG1 antibodies that, compared to IgG2 antibodies, are poor opsonins.

## Background

*Mycoplasma bovis *is an important cause of chronic pneumonia in feedlot cattle and dairy calves. Both in spontaneous and experimentally infected animals, different patterns of inflammatory lung lesions occur, among which caseonecrotic pneumonia is considered distinctive [1]. Findings in spontaneously occurring *M. bovis *infections suggest that necrotic lesions originate from bronchioles or small bronchi [2]. The chronicity of lung lesions and the persistence of *M. bovis *implies that the immune response is insufficient in eliminating the pathogen [2,3]. However, the mechanisms leading to tissue damage and how *M. bovis *evades the host immune response are incompletely understood [1,4]. The factors of *M. bovis *potentially associated with virulence are the variable surface membrane proteins (Vsps) [5]. In addition, other surface proteins, unrelated to the Vsps, e.g. pMB67, have been described [6-8]. Variable expression of these proteins may be a major mechanism by which *M. bovis *evades the immune response [1]. In a previous report the *in vivo *expression of Vsp antigens in lung tissue of calves inoculated with a clonal variant of *M. bovis *type strain PG45 by using immunohistochemistry (IHC) and different monoclonal Vsp-specific antibodies during early postinfectious stages, i.e. between 2 and 10 days post inoculation (p.i.) was described [9]. So far, it is not known if Vsp antigens are still present during the chronic stages of pneumonic lesions induced by *M. bovis*.

There are several reports, in which the humoral and cellular immune responses, i.e. presence of antibodies in sera and tracheobronchial lavage fluid, and *in vitro *stimulation and cytokine production of peripheral T lymphocytes, in spontaneous or experimentally *M. bovis *infected cattle was studied [10-12]. Pneumonic lesions in *M. bovis-*infected animals are usually accompanied by proliferation of the bronchus-associated lymphoid tissue (BALT) collectively known as "cuffing pneumonia" [2,3,13,14]. There is, however, only limited information about the types of cells involved of the immune response in lungs of *M. bovis *infected cattle [10-12,15,16].

In this investigation, the lungs of eight calves were examined three weeks p.i. with *M. bovis *strain 1067. One aim was to further characterize the pathology of experimentally induced lung lesions. The second aim was to examine the presence of Vsp antigens within lung tissue and to correlate the findings with local immune responses, i.e. immunoglobulin-containing plasma cells, CD4^+ ^and CD8^+ ^T lymphocytes, and expression of MHC class II.

## Methods

### Animals and experimental infection

For this study, lung tissue samples from eight experimentally infected male calves and four male control calves, all of the Simmental breed and originating from different *M. bovis *infection experiments were used. Before inoculation, tracheobronchial lavage fluid (TBLF) was taken from all calves to exclude the presence of *M. bovis *by bacteriological culture [17,18] and antibodies to *M. bovis *by ELISA [19,20]. The cultures were negative. In blood samples, *M. bovis*-specific serum antibodies were not detected by ELISA. All calves were inoculated at the age of approximately four weeks by the intratracheal route with 30 ml of fresh culture containing 1 × 10^8 ^(Nos. 1 and 3), 1 × 10^10 ^(Nos. 2 and 4) or were inoculated endobronchially with the same volume of inoculum containing 7.4 × 10^9 ^(Nos. 5-8) colony forming units (CFU) per ml of *M. bovis *strain 1067 [19]. Before inoculation, calves were sedated by intramuscular injection of 0.05 mg xylazine (Rompun, Bayer, Austria) per kg body weight. *M. bovis *was inoculated with a 6 mm diameter fiberoptic bronchoscope (polydiagnost, Pfaffenhofen, Germany). Infected calves (Nos. 1-8) and control calves (Nos. 9-12) were housed in separate pens in the Institute of Bacteriology, Mycology and Hygiene at the University of Veterinary Medicine, Vienna, Austria, according to the Austrian Act for Animal Experiments. Negative control calves were inoculated intratracheally (Nos. 9 and 10) or endobronchially (Nos. 11 and 12) with sterile mycoplasma broth alone. All calves were examined clinically once every day throughout the experiment by measuring the body temperature, respiratory rate, pulse rate, and by auscultating heart and lung.

Approval of the animal experiments was given by the Austrian Bundesministerium für Wissenschaft und Verkehr (registration numbers: 68.205/78-Pr/4/1998 and 68.205/29-Pr/4/2000).

### Necropsy and sampling

Twenty one days p.i. all animals were euthanised with sodium pentobarbitone and submitted for necropsy. Lung samples were collected from all calves for cultural isolation of *M. bovis *as previously described [20-22]. For isolation of other bacteria, samples were plated on Columbia agar (Oxoid, Basingstoke, UK) with 5% sheep blood and incubated at 37°C in 5% CO_2_. Identification of bacterial isolates was performed using standard identification methods. For histology and IHC, lung samples were collected from six standardized regions of the anterior, posterior cranial, and caudal lobes from both left and right lungs. From each of the six regions, two lung samples were fixed in 4% neutral-buffered formalin, processed and embedded for histology and IHC and a third sample was embedded in Tissue Tek (OCT compound, Sakura, Finetek Europe BV, Alphen aan den Rijn, The Netherlands), placed in liquid nitrogen and then stored at -70°C until examined by IHC. In case of grossly detectable lesions, one sample was taken from an area with lesions and the other from a macroscopically unremarkable area. Both samples were fixed in 4% formalin. For culturing, two samples each from areas with gross lesions of the right and left lung were collected.

### Histopathology

Formalin-fixed samples were embedded in paraffin wax, sectioned and stained with haematoxylin and eosin (H&E). On selected sections, i.e. sections with necrotic lesions, Gram stain was used as well.

### Immunohistochemistry

The different antibodies and details of their application are given in Table 1. IHC for *M. bovis *antigens on paraffin sections was carried out as previously described [9]. As positive controls for detection of immunoglobulins, T lymphocytes and MHC class II sections of normal bovine lymph node tissue were used. As positive control for staining of *M. bovis *antigen, lung sections from a calf from another experiment, which had been euthanised after respiratory infection with *M. bovis *strain 1067, were used. For all immunohistochemical reactions, the Avidin:Biotinylated enzyme Complex (ABC) method was applied. In negative control sections the primary antibodies were replaced by normal mouse (BALB/c) serum (BioLogo, Kronshagen, Germany), normal sheep or normal rabbit serum, respectively, diluted in phosphate-buffered saline (pH 7.2, 0.15 M) using the same dilution as for the primary antibodies.

**Table 1:** Antibodies used for immunohistochemistry on paraffin and/or frozen sections

Antibody **Clone/designation**^**1**^	**Working dilution**^**2**^	Type or isotype	**Specificity**^**3**^	**Antigen retrieval**^**4**^	Source/Reference
mAbpool	1:2000^a^	Mouse IgG1 and IgM	*M. bovis*	0.25% trypsin (37°C, 60 min)	Chemicon, Temecula, CA, USA
1A1	1:200^a^/1:800^b^	Mouse IgG1	*M. bovis *Vsp^a ^A, C	0.25% trypsin (37°C, 60 min)	[46]
1E5	u.d.^c^/1:70^b^	Mouse IgM	*M. bovis *Vsp^a ^A, B, C	0.25% trypsin (37°C, 60 min)	[47]
I_2_	1:900^b^	Mouse IgG1	*M. bovis *pMB67^b^	w/o^a^	[7,48]
CC30	1:5^b^	Mouse IgG1	Bovine CD4	w/o^a^	Biozol, Eching, Germany
CC63	1:50^b^	Mouse IgG2a	Bovine CD8	w/o^a^	Biozol, Eching, Germany
A10-116F^a^	1:8000^a^	Sheep IgG	Bovine IgG1	0.05% pronase E (37°C, 20 min)	Bethyl Laboratories, Montgomery, TX, USA
A10-117^a^	1:3000^a^	Sheep IgG	Bovine IgG2	0.05% pronase E (37°C, 20 min)	Bethyl Laboratories, Montgomery, TX, USA
A10-100F^a^	1:400^a^	Rabbit IgG	Bovine IgM	0.05% pronase E (37°C, 20 min)	Bethyl Laboratories, Montgomery, TX, US
A10-108F^a^	1:4000^a^	Rabbit IgG	Bovine IgA	0.05% pronase E (37°C, 20 min)	Bethyl Laboratories, Montgomery, TX, USA
AE1/AE3	1:500^a^	Mouse IgG	Wide spectrum-cytokeratin	0.05% pronase E (37°C, 20 min)	DakoCytomation, Hamburg, Germany
TAL.1B5	1:4000^a^	Mouse IgG1	α-chain of human leukocyte antigen (HLA-DR)	0.01 M citric buffer (microwave 95°C, 15 min)	DakoCytomation, Hamburg, Germany

^1a ^Conjugated to fluorescein isothiocyanate (FITC).

^2a ^On paraffin sections. ^2b ^On frozen sections. ^c ^Antibody was used undiluted.

^3a ^Variable surface membrane protein of *M. bovis*. ^b ^Non-Vsp surface antigen of *M. bovis*.

^4a ^Without antigen retrieval.

### Quantitative evaluation of T lymphocytes and plasma cells

For CD4^+ ^and CD8^+ ^cells, 1000 cells were counted within the BALT of bronchioli in all six frozen samples of each animal by light microscopy and the number of positively reacting cells was determined. The number of positive cells was then calculated as mean values per μm^2 ^of BALT area. The number of immunoglobulin-containing plasma cells per 1 mm^2 ^of BALT of small bronchi and bronchioli was determined with a computer image analysis system (AnalySIS 3.1, Olympus Soft Imaging Solutions, Münster, Germany) at × 200 magnification. A comparative analysis of the number of CD4^+ ^and CD8^+ ^cells and plasma cells containing the different immunoglobulins in control and inoculated animals was performed with the non-parametric Mann-Whitney-U-test. The level of statistic significance was set at *P *< 0.05.

## Results

### Clinical findings

After inoculation, three calves (Nos. 3, 5, 8) had increased rectal temperatures with mean values ranging between 39.3 and 40.1°C. In these three calves, an increased respiratory rate was recorded exceeding 60/min (No. 8) or 70/min (Nos. 3 and 5). Furthermore, these three animals showed nasal discharge, coughing and reduced appetite until euthanasia. In the other calves and in all control calves the rectal temperature and the respiratory rate were normal.

### Bacteriology

Except from one calf (No. 8), *M. bovis *was detected by cultural isolation in lung samples from all other inoculated animals. From lung samples of six calves other bacteria were isolated and identified as *Arcanobacterium pyogenes *(Nos. 5 and 6), *Pasteurella multocida *(Nos. 3 and 4), α-haemolytic streptococci and *Staphylococcus aureus *(Nos. 1-4), and enterococci (No. 1). Cultural examination of the lungs from control calves was negative for *M. bovis *but positive for α-haemolytic streptococci, *S. aureus *and enterococci in two (Nos. 9 and 10).

### Macroscopical findings

**Figure 1:**
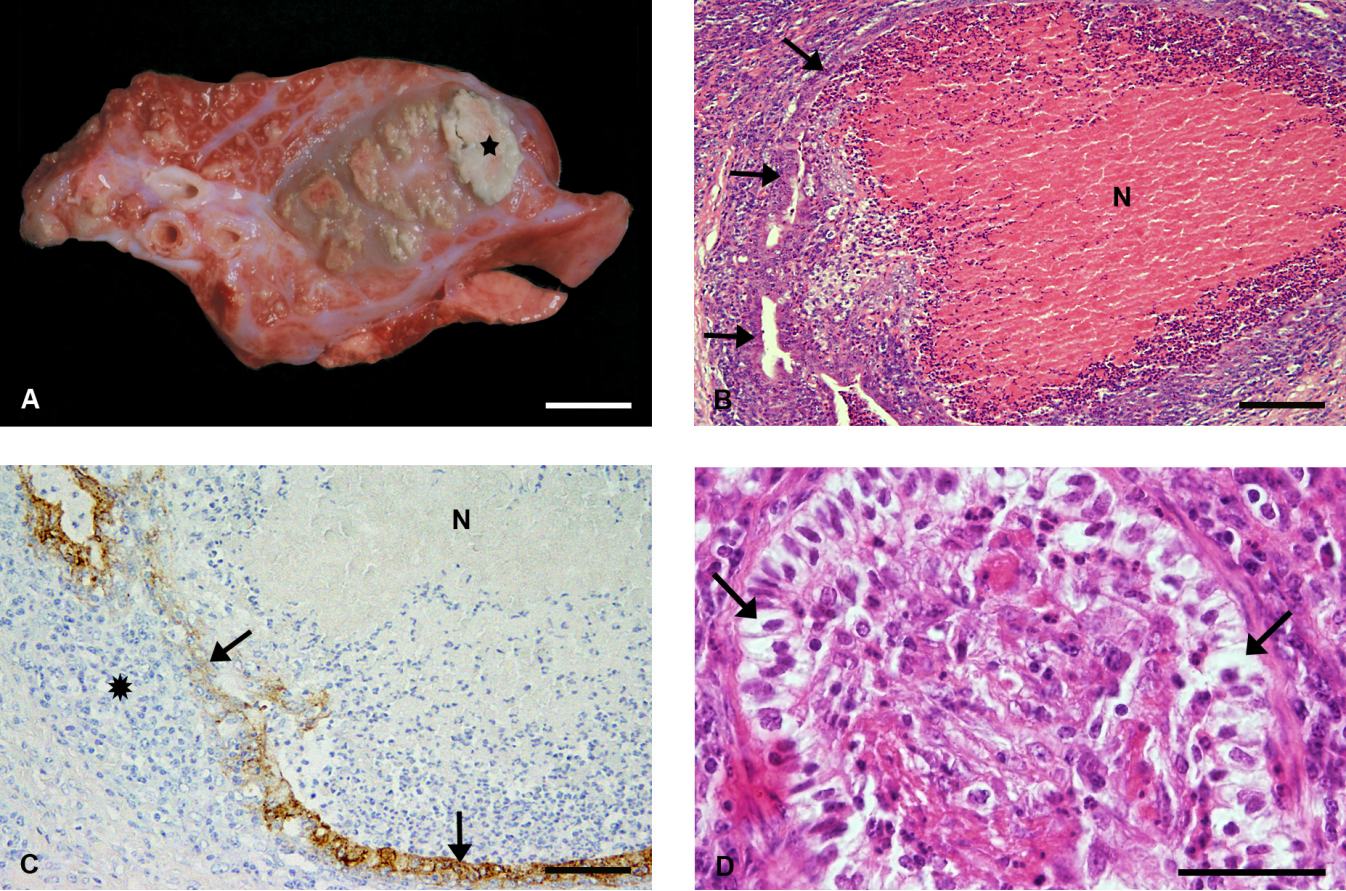
**(A) Gross pathology of caseonecrotic pneumonia; cut surfaces of lung tissue with various sized caseonecrotic lesions (largest area depicted by asterisk)**. There is marked interlobular fibrosis and suppurative bronchopneumonia in the remaining lung parenchyma. Calf no. 5. Bar = 1 cm; (B) Histopathology of caseonecrotic pneumonia; caseonecrotic lung lesion. The eosinophilic centre (N) is demarcated by inflammatory cells and remnants of necrotic bronchiolar epithelium (arrows). Calf No. 3. H&E. Bar = 184 μm; (C) Immunohistochemical detection of wide spectrum cytokeratin-positive epithelial cells; immunohistochemical staining with antibodies (mAb AE1/AE3) to wide spectrum cytokeratin of the same bronchiole as shown in (B) reveals remnants of bronchiolar epithelial cells (arrows) surrounding the necrotic area (N). At the periphery, demarcating macrophages are present (asterisk). Calf No. 3. ABC method. Bar = 92 μm; (D) Histopathology of obliterative bronchiolitis; obliterative bronchiolitis with vacuolated epithelial cells (arrows) and intraluminal fibroblasts and inflammatory cells. Calf No. 5. H&E. Bar = 46 μm.

In three inoculated animals (Nos. 4, 6 and 7), white exudates drained off from small bronchi of the cranial lung lobes, consistent with slight suppurative bronchitis, affecting less than 10% of total lung surface. The lungs of the other five inoculated calves had varying degrees of consolidation of the apical lung lobes (Nos. 1, 2, 3, 5, 8). Two of these animals (Nos. 3 and 5) had marked caseonecrotic pneumonia with several foci of approximately up to 0.5 cm (No. 3) or up to 5.0 cm (No. 5) of white-yellow, friable, caseous material, which was frequently surrounded by pale firm connective tissue (Figure 1A). In five calves (Nos. 1, 2, 3, 5, 8) between 10 and 50% of total lung surface was affected. In all eight animals, macroscopic changes were located in the left and/or right apical lung lobes, and in the majority of lungs the middle lobe was also affected. Lesions in the diaphragmatic lobes were only found in calves which had severe lung lesions such as consolidation, and/or necrosis.

The lungs of three control calves were macroscopically unremarkable. One control animal (No. 10) had slight consolidation in one apical lung lobe.

### Histopathology and immunohistochemistry for wide spectrum cytokeratin

The histological lung lesions in inoculated calves are summarised in Table 2. In all inoculated calves, interstitial pneumonia in combination with suppurative bronchopneumonia and/or suppurative bronchitis and bronchiolitis, were found. In two calves, marked caseonecrotic pneumonia (Figure 1B-D) and multifocal obliterative lesions of bronchioli were present. Caseonecrotic areas were of variable size and their centres contained eosinophilic material and small amounts of debris. They were surrounded by accumulations of mostly degenerate neutrophilic granulocytes, macrophages and an outer zone of plasma cells and lymphocytes. In the surrounding lung parenchyma bronchioli with focal or complete necrosis of the epithelium were frequently seen. In sections stained for wide spectrum cytokeratin from different lung lobes of the two calves with caseonecrotic pneumonia, positive epithelial cells resembling remnants of bronchiolar, bronchial and peribronchial gland epithelium were found (Figure 1C).

**Table 2:** Histopathological lung lesions in calves inoculated with *Mycoplasma bovis *strain 1067 and in control calves

Calf **No**.	Bronchointerstitial **pneumonia**^**1**^	Suppurative bronchitis and bronchiolitis	Suppurative bronchopneumonia	Caseonecrotic pneumonia	Obliterative Bronchiolitis	BALT hyperplaisia
1	+^a^	+^a^	+/++^d^	-^c^	-^c^	++^b^
2	+^a^	+^a^	+/++^d^	-^c^	-^c^	++/+++^e^
3	+^a^	-^c^	++/+++^e^	++/+++^e^	+++^f^	++/+++^e^
4	++^b^	+^a^	-^c^	-^c^	-^c^	+++^f^
5	+^a^	+^a^	+++^f^	+++^f^	+++^f^	++/+++^e^
6	+^a^	+^a^	-^c^	-^c^	-^c^	+/++^d^
7	+^a^	+^a^	-^c^	-^c^	-^c^	+/++^d^
8	+^a^	-^c^	++^b^	-^c^	++^b^	++/+++^e^
9	+^a^	+^a^	-^c^	-^c^	-^c^	+/++^d^
10	+^a^	-^c^	+^a^	-^c^	-^c^	+/++^d^
11	+^a^	-^c^	+^a^	-^c^	-^c^	+/++^d^
12	+^a^	-^c^	+^a^	-^c^	-^c^	+/++^d^

^1^Infiltration of alveolar septa with macrophages, lymphocytes and neutrophilic granulocytes.

^a^Mild. ^b^Moderate. ^c^None. ^d^Mild to moderate. ^e^Moderate to severe. ^f^Severe.

Obliterated bronchioli often had vacuolated epithelial cells and accumulations of neutrophilic granulocytes within their lumen (Figure 1D). Often partial to nearly complete loss of bronchiolar epithelial cells was present. Within the lumen of such obliterated bronchioles, tissue masses composed of fibroblasts and collagen fibres were found. In most locations, macrophages and sometimes a few neutrophilic granulocytes, were seen within these intraluminal tissue masses.

In one animal (No. 5), beside caseonecrotic pneumonia, there was a focus of coagulation necrosis, which was surrounded by numerous degenerate leukocytes, but without the presence of so-called oat cells. The outlines of alveoli were still visible and there were Gram-positive bacteria within the centre of the coagulation necrosis.

Between lung tissue samples from intratracheally or endobronchially inoculated animals no differences were found.

The lungs of all control calves had minimal to sometimes mild infiltration of the alveolar septae of apical lobes with macrophages, lymphocytes and few neutrophilic granulocytes, accompanied by mild focal suppurative bronchitis and bronchiolitis. The consolidated areas of one apical lobe of control calf No. 10 had mild suppurative bronchopneumonia.

### Immunohistochemistry for *M. bovis *antigen

The distribution of *M. bovis *antigen is summarised in Table 3. In all calves, macrophages, i.e. alveolar macrophages, macrophages in alveolar septae and macrophages within lumina of bronchi and bronchioli containing an inflammatory exudate, had antigen within their cytoplasm. In the cytoplasm of neutrophilic granulocytes, antigen was less frequently found. With the mAb pool a fine granular staining of antigen was associated with exudate in the lumina of bronchi and bronchioli. Positive labelling with this antibody pool was also found on the surfaces of epithelial cells in larger airways and sometimes also on the surfaces of alveoli and within alveolar septae. In sections from the two calves with caseonecrotic pneumonia, accumulations of fine granular extracellular antigen mainly located in the peripheral zones of the necrotic areas, were seen. In calf No. 5, no differences in the amount and distribution of *M. bovis *antigen between the caseonecrotic foci and the area of coagulation necrosis were found. In sections from calf No. 5 with obliterative bronchiolitis, there were a few positive neutrophilic granulocytes infiltrating the fibrous tissue obliterating a single bronchiole.

**Table 3:** Distribution of *M. bovis *antigens in lungs of 8 experimentally inoculated calves

**Location**^**1**^	**mAb pool**^**2**^	**mAb 1A1**^**3**^	**mAb 1E5**^**4**^	mAb I_2_^5^
Macrophages^a^	8/8^a^;8/8^b^	8/8^a^;8/8^b^	0/8^a^;6/8^b^	NR^a^;5/8^b^
Exudate in lumina of large airways	8/8^a^;8/8^b^	6/8^a^;6/8^b^	0/8^a^;4/8^b^	NR^a^;4/8^b^
Epithelial surface of large airways	6/8^a^;6/8^b^	4/8^a^;4/8^b^	0/8^a^;2/8^b^	NR^a^;0/8^b^
Alveolar surface	3/8^a^;3/8^b^	1/8^a^;1/8^b^	0/8^a^;2/8^b^	NR^a^;0/8^b^
Caseous necrosis	2/2^a^;-^c^	2/2^a^;-^c^	0/8^a^;-^c^	NR^a^;-^c^

^1a^Alveolar macrophages, macrophages in alveolar septa and macrophages within lumina of bronchi and bronchioli.

^2a,3a,4a ^Number of positive calves/number of calves examined (paraffin sections).

^2b,3b,4b,5b ^Number of positive calves/number of calves examined (frozen sections).

^2c,3c,4c,5c ^Necrotic lesions were not present in lung samples, from which the frozen sections were prepared.

^5a ^No reactivity.

**Figure 2:**
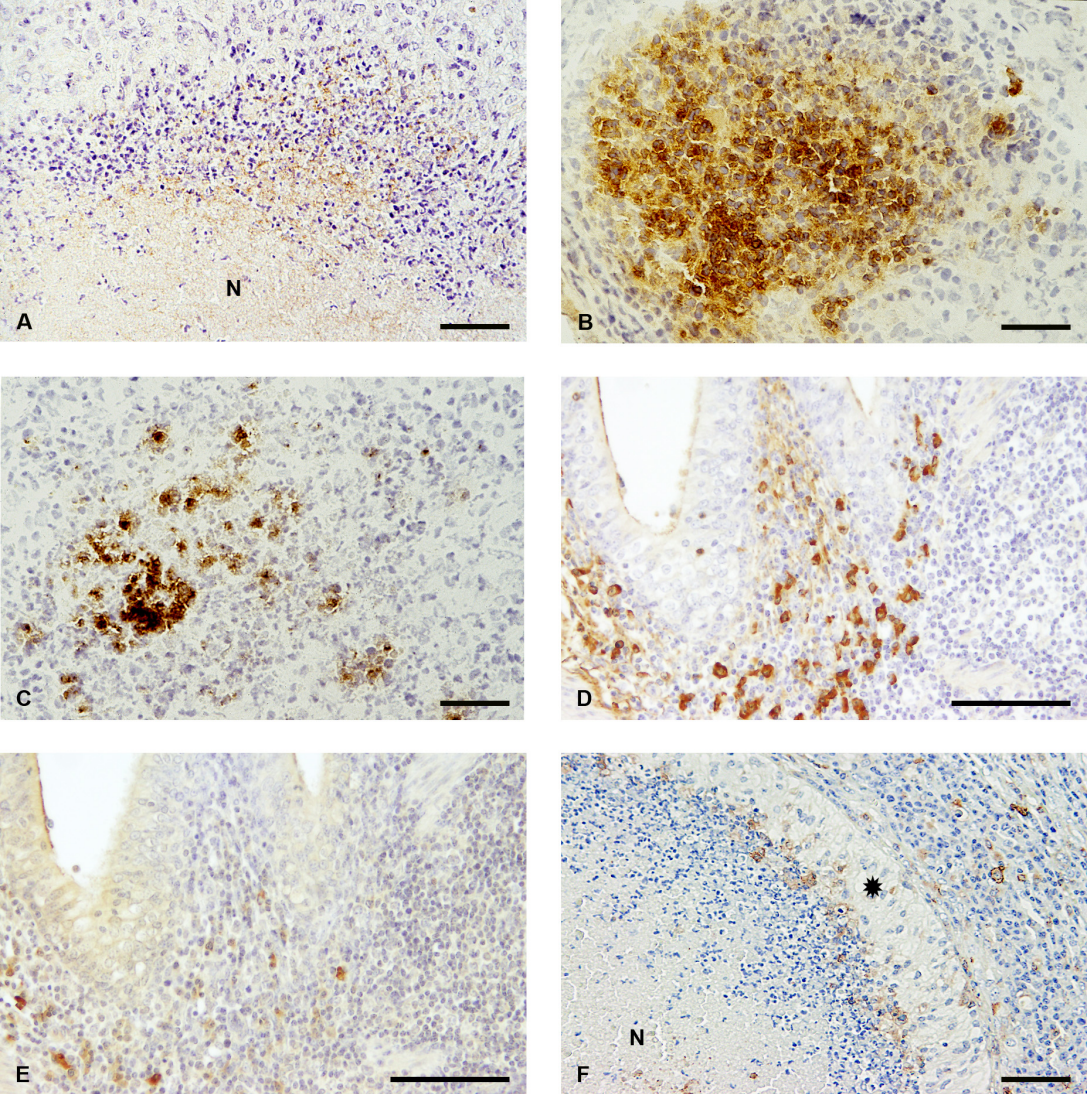
**(A) Immunohistochemistry for *M. bovis *Vsp antigen in necrotic lesion; the centre of a caseonecrotic pulmonary lesions (N) is surrounded by mostly degenerate neutrophilic granulocytes and macrophages**. Immunohistochemical labelling of extracellular accumulations of *M. bovis *antigens with mAb 1A1. Calf No. 5. ABC method. Bar = 46 μm; (B) Immunolabelling of *M. bovis *Vsp antigens with mAb 1A1 within the lumen of a bronchiole containing exudate. Calf No. 3. ABC method. Frozen section. Bar = 46 μm; (C) Immunolabelling of *M. bovis *variable antigen pMB67 with mAb I2. Sequential frozen section of the same location as in (B); (D) Numerous IgG1-positive plasma cells in the lamina propria of bronchial mucosa. Calf No. 8. ABC method. Bar = 92 μm; (E) Lower numbers of IgG2-positive plasma cells in the lamina propria of bronchial mucosa in a sequential paraffin section of the same location as in (D); (F) Immunohistochemistry for MHC class II in necrotic and perinecrotic lung area; caseonecrotic lung lesion with MHC class II immunoreactivity within the necrotic area (N) and within the perinecrotic region. The majority of macrophages surrounding the necrosis (asterisk) is negative for MHC class II. Calf No. 5. ABC method. Bar = 92 μm.

In sections stained with mAb 1A1, Vsp antigens showed a similar distribution pattern as detected with the mAb pool (Figure 2A and 2B). Vsp antigens were found in macrophages of all calves. In other locations, however, positive staining of Vsp antigens was less frequently present than positive staining with the mAb pool (Table 3). With mAb 1E5, immunoreactivity was only seen on frozen sections. The distribution of Vsp antigens was similar to the staining pattern received with mAb 1A1, although positive reactions were found less frequently. With mAb I_2_, *M. bovis *antigen pMB67 was only detected in the cytoplasm of macrophages and within the exudate in small bronchi and bronchioli. Occasionally, co-localization of variable antigens was observed with mAbs 1A1 and I_2 _in sequential sections of necrotic areas and within the lumen of small bronchi and bronchioli (Figure 2B and 2C). Lung sections from the control calves were negative.

### Immunohistochemistry for CD4^+ ^and CD8^+ ^T lymphocytes

The total numbers of CD4*^+ ^*and CD8*^+ ^*T cells are given in Table 4. Statistical analysis did not reveal a significant difference for CD4*^+ ^*or CD8^+ ^T lymphocytes in hyperplastic BALT between inoculated and control calves. CD4*^+ ^*T cells were mainly located in perifollicular areas of the BALT. Few CD4*^+ ^*T cells were found in the subepithelial tissue of small bronchi and bronchioli. Occasionally, CD4*^+ ^*T cells were seen within the epithelium of small bronchi and bronchioli and in alveolar septae. CD8*^+ ^*T lymphocytes were mainly located in alveolar septae whereas only scattered cells were present in the perifollicular areas of the BALT.

**Table 4:** Total numbers of peribronchially located CD4^+ ^and CD8^+ ^T lymphocytes and numbers of immunoglobulin-positive plasma cells

**Calf No.**^**1**^	CD4^+ ^T cells^2^	CD8^+ ^T cells^3^	**IgA**^**4**^	**IgG1**^**5**^	**IgG2**^**6**^	**IgM**^**7**^
1	12.40 ± 3.19^a^	1.10 ± 1.72^a^	536^a^	698^a^	305^a^	250^a^
2	6.60 ± 3.46^a^	0.40 ± 0.16^a^	634^a^	343^a^	236^a^	195^a^
3	15.3 ± 6.16^a^	2.10 ± 0.98^a^	314^a^	421^a^	160^a^	245^a^
4	10.40 ± 2.50^a^	1.30 ± 1.30^a^	240^a^	723^a^	167^a^	294^a^
5	13.30 ± 7.01^a^	1.00 ± 1.00^a^	530^a^	484^a^	479^a^	293^a^
6	8.80 ± 1.76^a^	ND^b^	760^a^	437^a^	339^a^	409^a^
7	11.40 ± 7.26^a^	0.20 ± 0.13^a^	695^a^	257^a^	227^a^	310^a^
8	8.80 ± 7.15^a^	1.20 ± 1.12^a^	431^a^	467^a^	297^a^	387^a^
9	2.00 ± 1.84^a^	0.20 ± 0.23^a^	528^a^	218^a^	100^a^	225^a^
10	10.20 ± 3.88^a^	2.00 ± 2.45^a^	497^a^	300^a^	110^a^	196^a^
11	10.10 ± 5.15^a^	0.40 ± 0.04^a^	482^a^	485^a^	262^a^	240^a^
12	-^b^	-^c^	464^a^	268^a^	223^a^	345^a^

^1 ^Calves Nos. 1-8 were inoculated with *M. bovis*, calves Nos. 9-12 were control calves.

^2a,3a ^Values given as means ± SD of the total numbers of positive cells counted per 1000 μm^2 ^of BALT area.

^2b,3c ^No BALT in tissue sections present.

^3b ^Not determined.

^4a ^IgA-positive plasma cells.

^5a ^IgG1-positive plasma cells.

^6a ^IgG2-positive plasma cells.

^7a ^IgM-positive plasma cells.

^4a,5a,6a,7a ^Values given as means of absolute numbers of positive cells per mm^2 ^of BALT.

### Expression of immunoglobulins

In both inoculated and control calves, the highest mean value of each group was found for IgA-positive plasma cells, followed by IgG1-positive plasma cells. The numbers of immunoglobulin-containing plasma cells are given in Table 4. Plasma cells expressing IgG2 or IgM were less frequently detected. The most pronounced differences were found in the apical lobes. Whilst the increase in IgA (252 to 298 cells per mm^2^) and IgM (493 to 517 cells per mm^2^) containing plasma cells was mild, considerably increased numbers of IgG1 (318 to 479 cells per mm^2^) and IgG2 (174 to 276 cells per mm^2^) containing cells were found by comparing the mean values of inoculated and control calves (Figure 2D and 2E). Level of significance, however, was not reached.

### MHC class II immunohistochemistry

In sections from all inoculated calves strong MHC class II immunoreactivity was seen on lymphoid cells in hyperplastic BALT and on cells in alveolar septae. The positivity of the respiratory epithelia of the bronchi and bronchioli varied. In areas with interstitial pneumonia and suppurative bronchopneumonia the respiratory epithelium adjacent to hyperplastic BALT was strongly positive whilst the respiratory epithelium of bronchi and bronchioli in the neighbourhood of caseonecrotic lesions was only weakly positive or negative. The epithelium of the majority of obliterated bronchioli was negative (Nos. 3 and 8) or partially positive (No. 5). Intra-alveolar macrophage immunoreactivity was either positive or negative. Perinecrotic areas had MHC class II-positive cells, but the majority of perinecrotic located macrophages were negative (Figure 2F). Within several necrotic areas a positive reaction was seen (Figure 2F). In sections of inoculated calves with interstitial pneumonia and suppurative bronchopneumonia numerous MHC class II positive cells with dendritic morphology were found, mainly located in the subepithelial tissue of the bronchial mucosa. In areas with caseonecrotic lesions and obliterative bronchiolitis only a few MHC class II expressing cells with dendritic morphology were seen. In sections from control calves, few MHC class II positive cells were present in alveolar septae. Furthermore, respiratory epithelial cells were weakly positive or negative and a few MHC class II positive cells with dendritic morphology were seen in the bronchial mucosa.

## Discussion

The results of this study show that inoculation with *M. bovis *strain 1067 causes caseonecrotic pneumonic lesions that originate from small bronchi and bronchioli. The possible mechanisms, however, by which *M. bovis *induces these lesions, are not clear. Whether *M. bovis *infects airway epithelial cells is controversial [1] and recent findings in experimentally infected animals suggest that positive immunohistochemical staining with antibodies to *M. bovis *seen in airway epithelial cells is non-specific. Therefore, beside direct effects of *M. bovis*, certain factors released by the host's lung tissue could be involved in the development of necrotizing lesions in large airways. A recent study of lung sections of the calves examined in this investigation revealed the co-localization of *M. bovis *antigen and of strongly expressed inducible nitric oxide and nitrotyrosine by macrophages in perinecrotic tissue areas [23] indicating that the production of nitric oxide and peroxynitrite is potentially involved in the development of necrotizing lung lesions. Increasing concentrations of peroxynitrite lead to the generation of reactive oxygen and nitrogene species (ROS and RNS) which both have cytotoxic capacities. Therefore, both ROS and RNS are potentially involved in the development of severe necrotizing lung lesions seen in the animals of this study.

Obliterative bronchiolitis was seen in three inoculated calves of this investigation. The occurrence of bronchiolitis obliterans in animals naturally infected with *M. bovis *has been described by other investigators [2,13,24]. Furthermore, bronchiolitis obliterans has been reported in calves with chronic pneumonic lesions associated with spontaneous and experimental infections with other bacteria, e.g. *Mannheimia haemolytica, P. multocida, Histophilus somni *and *Mycoplasma dispar*, and with bovine respiratory syncytial virus [25-29]. The obliterative changes seen in *M. bovis*-infected calves resemble lesions classified as "bronchiolitis obliterans with intraluminal polyps" in man [30], which occur in cases of organizing pneumonia in humans and are known as "bronchiolitis obliterans organizing pneumonia" (BOOP). Organizing lesions in the alveolus, i.e. alveolar fibrosis, as described for BOOP in man [31] were not present in the calves with obliterative bronchiolitis of this study, but were described in calves spontaneously infected with *M. bovis *[2]. Therefore, and also because re-epithelization of fibrous tissue within affected bronchioli was not present, the obliterative changes found in the bronchioli of the three calves of the present study might represent an early stage of organization. Recent studies on the lung tissue from these three calves demonstrated increased production of inducible nitric oxide and nitrotyrosine suggesting that nitric oxide and peroxynitrite are potentially involved in the development of obliterative bronchiolitis [23].

In a previous study, we demonstrated *in vivo *expression of *M. bovis *Vsps in lung tissue of calves infected with a clonal variant of *M. bovis *type strain PG45 during the first ten days p.i. [9]. The present investigation revealed that there is long-term persistence of *M. bovis *in chronic bronchopneumonic lesions as demonstrated by bacteriology and IHC for antigens, i.e. Vsp antigens and pMB67.

The distribution of Vsp and non-Vsp antigens of *M. bovis *found in this study closely resembles the pattern described by other investigators who used different poly- and/or monoclonal antibodies to *M. bovis *[2,3,14]. With mAb 1A1, apart from the positive macrophages, positive reactions for Vsp antigens and also for the antigen pMB67 occurred less frequently than with the mAb pool. A possible reason for this is that the mAb pool detects both variable and non-variable antigens. A constant finding in lungs of all inoculated calves in this study was the presence of *M. bovis *Vsp and non-Vsp antigens in the cytoplasm of macrophages. This suggests that *M. bovis *is taken up by phagocytosis following opsonisation and that residual antigen, possibly after killing of the organism, persists detected by IHC. Another possibility would be that whole organisms of *M. bovis*, after being phagocytosed, survive within the phagosome of macrophages.

*In vitro *studies have shown that, except from variable surface antigens recognized as potential virulence factor of persistence in the host, *M. bovis *is able to generate a biofilm [32]. Further studies to determine if biofilms also occur *in vivo*, i.e. on the surfaces of the respiratory tract in *M. bovis *infected calves, and if or how they contribute to the persistence of the agent in the host, are necessary.

In all *M. bovis *infected calves, hyperplasia of BALT was characterized by strong MHC class II expression of lymphoid cells within the BALT. This finding indicates ongoing stimulation of the local pulmonary immune system in response to persisting *M. bovis *antigen. Only few of the macrophages demarcating the caseonecrotic foci were positive for MHC class II, further supporting the hypothesis that, although *M. bovis *antigen is still present in necrotizing lesions, the antigen-presenting mechanisms are down-regulated at chronic stages of the disease. Nitric oxide is known to play a role as a modulator of immune responses. Therefore, the low expression of MHC class II by macrophages in perinecrotic areas of *M. bovis *infected calves reported by other investigators [33] and also seen in this study, possibly represents down-regulation of MHC class II-mediated antigen presentation as a result of the production of inducible nitric oxide and nitric oxide by activated macrophages. Beside macrophages, pulmonary dendritic cells play an important role in antigen presentation and induction of T cell-mediated immune responses in the lung [34]. A previous study, in which quantification of MHC class II expressing dendritic cells in calves examined in this study was carried out, showed that statistically significantly increased numbers of MHC class II-expressing dendritic cells were present in the mucosa of bronchi and bronchioli of *M. bovis *infected animals [35]. In this study, examination of lung sections revealed that, in caseonecrotic foci and obliterated bronchioli, in contrast to the respiratory mucosa, only few MHC class II expressing dendritic cells were present, possibly indicating down-regulation of antigen presentation in these areas.

Reduced numbers of MHC class II expressing dendritic cells could be the result of the production of inducible nitric oxide and nitric oxide by activated macrophages. Otherwise, lesser expression of MHC class II could be a non-specific consequence of chronic immunostimulation reflecting lower amounts of MHC class II-inducing cytokines, e.g. IL-1 and IFN-γ, at the chronic stage of the disease.

Experimental infections of calves have shown that *M. bovis *has both stimulating and suppressing effects on the bovine immune response such as stimulating the production of nitric oxide and TNF-α by macrophages, inducing apoptosis of lymphocytes, producing a lympho-inhibitory peptide, impairing lymphocyte responses to mitogens and suppressing the neutrophil oxidative burst [36-39].

Aside from a few studies [11,12], only limited information is available on the local immune response in lung tissue of calves infected with *M. bovis *[10,15,16].

The present study revealed a considerable increase of IgG1- and IgG2-positive plasma cells at 21 days p.i. among which IgG1-containing plasma cells clearly predominated. This finding is consistent with the results previously obtained by Howard *et al. *[10]. In one study [12], increased IgG1 antibodies were found in the sera of experimentally infected cattle, but only small amounts of IgG2 antibodies. The authors concluded that the immune response mounted against *M. bovis *infection was skewed toward a T helper 2 immune response. It has been speculated by others [12] that, because IgG2, in comparison to IgG1, is the superior opsonin, the low IgG2 response may contribute to the chronicity of *M. bovis *infection. *In vitro *studies with bovine alveolar macrophages and bovine polymorphonuclear leukocytes indicate that opsonisation, i.e. specific sera, promote phagocytosis and killing of *M. bovis *by phagocytes [40].

The immunohistochemical finding of many antigen-positive macrophages suggests that phagocytosis, possibly opsonophagocytosis, does occur *in vivo*. However, because in necrotic foci high amounts of extracellular antigen are found adjacent to phagocytes, the process of phagocytosis could be modified during the course of infection by yet unknown mechanism. Differentiation of B cells into antibody secreting plasma cells usually is due to cytokine secretion by helper function of CD4^+ ^T lymphocytes. Statistical evaluation of the numbers of CD4^+ ^and CD8^+ ^T cells in this study, however, did not reveal statistically significant differences between inoculated and control calves.

In this study, *M. bovis *was isolated from the lungs of 7 of 8 experimentally infected calves at necropsy. In the lungs of all calves inoculated with *M. bovis*, suppurative inflammatory changes of bronchi and bronchiole, often associated with suppurative bronchopneumonia, were found. Since pyogenic bacteria were isolated from the majority of these calves, they are possibly responsible for the development of the suppurative lung lesions. These results agree with the findings of other investigators that *M. bovis *is a predisposing factor in bovine respiratory disease allowing colonization of the lower respiratory tract by commensal pathogenic bacteria [3,41]. Although caseonecrotic pneumonia is considered to be a distinctive lesion caused by *M. bovis *[1], the present findings support the hypothesis of other investigators that severe caseonecrotic lesions mainly occur when other bacteria are present [42]. Co-infection of calves after spontaneous or experimental infection with *M. bovis *has also been described by other investigators [1]. One report, in which the same *M. bovis *field strain as in this study was used, describes co-occurring *P. multocida *infection in 10 of 16 conventionally reared experimentally infected calves [43]. The polymicrobial infection being present at 21 days p.i. in the calves of this study is complicating the interpretation of the role of *M. bovis *in the development of the local immune response in the lungs of infected calves. It cannot be excluded that the other bacteria isolated at the end of the experiment together with *M. bovis *participated in the generation of the immune response in these animals.

In lung tissue of the control calves, which were microbiologically negative for *M. bovis*, minimal or mild inflammatory changes including mild suppurative bronchitis and bronchiolitis, being associated with the presence of *S. aureus*, were seen. Therefore, it cannot be excluded that during the repeated manipulations necessary for collecting TBLF samples bacteria from the upper respiratory tract were flushed into the lungs of control calves animals and possibly also of *M. bovis *infected animals.

The three calves with caseonecrotic pneumonia and/or obliterative bronchiolitis had signs of clinical disease, i.e. increased body temperature and respiratory rate, nasal discharge, coughing and reduced appetite. In the other five calves, however, in spite of having lung lesions such as suppurative inflammatory changes of larger airways and/or suppurative bronchopneumonia, no clinical signs, i.e. increased body temperature, respiratory rate or pulse rate or abnormal findings by auscultating heart and lung, were recorded. These findings indicate that, at least under the experimental conditions of this study, respiratory *M. bovis *infections of calves can cause lung lesions, which, by applying conventional methods of clinical examination, are not associated with detectable signs of respiratory disease. The clinical signs and their presence in experimentally infected animals reported in the literature vary concerning type of signs and number of animals showing such signs per experiment. In one report, the occurrence of subclinical pneumonia, i.e. the absence of clinical signs of respiratory disease in spite of lung lesions was recorded in nine of ten calves 14 days after inoculation with *M. bovis *[3]. These findings are similar to our observations in five of eight of the inoculated animals. Clinical signs associated with bovine respiratory disease vary and signs may be minimal or absent in cases with minor and/or chronic lung lesions [44]. Other methods such as radiology, ultrasonography and lung function testing are considered as useful techniques for diagnosing clinically silent pneumonic lesions and for correlating clinical signs with pathological findings [44,45].

## Conclusions

Our findings show that infection of calves with *M. bovis *strain 1067 results in various lung lesions including caseonecrotic pneumonia. IHC for wide spectrum cytokeratin in two calves with caseonecrotic foci demonstrated that these lesions originated from bronchioli and bronchi. Our results show that there is long-term persistence of *M. bovis *as demonstrated by bacteriology and IHC for *M. bovis *antigens, i.e. VSp antigens and pMB67. The persistence of the pathogen and its ability to evade the specific immune response may in part result from (i) local downregulation of antigen presenting mechanisms and (ii) an ineffective humoral immune response with prevalence of IgG1 antibodies that, compared to IgG2 antibodies, are poor opsonins.

## Competing interests

The authors declare that they have no competing interests.

## Authors' contributions

KH and MH-T have conducted the histopathology and drafted the manuscript. IB and BB have performed the IHC and evaluation of *M. bovis *Vsp antigens, CD4^+ ^and CD8^+ ^T cells and immunoglobulins in plasma cells and performed the statistical analysis. AT performed the IHC and evaluation of MHC class II. JS and RR have designed the bacteriological part of the study and analysis and interpretation of bacteriological results. All authors have revised the manuscript and approved the final manuscript.
